# Burn Pits Exposure and Chronic Respiratory Illnesses among Iraq and Afghanistan Veterans

**DOI:** 10.15436/2378-6841.19.2429

**Published:** 2019-02-09

**Authors:** Steven S. Coughlin, Anthony Szema

**Affiliations:** 1 Department of Population Health Sciences, Medical College of Georgia, Augusta University, Augusta, GA; 2 Research Service, Charlie Norwood VA Medical Center, Augusta, GA; 3 Occupational Medicine, Epidemiology, and Prevention, Donald and Barbara Zucker School of Medicine at Hofstra/Northwell, NY

## Introduction

Since the beginning of the current conflicts in Southwest Asia, more than 2 million service members have deployed in support of Operation Enduring Freedom (OEF) and Operation Iraqi Freedom (OIF) Sharkey (Sharkey et al. 2016). Service members can be exposed to a variety of environmental hazards during deployment including pollutants from unregulated industry, particulate matter from desert environments, exhaust from military vehicles, and emissions from open-air burn pits. Exposure to emissions from burn pits has been a cause for concern because of the potential for respiratory health conditions (asthma, bronchitis, chronic obstructive pulmonary disease, constrictive bronchiolitis) and other chronic health conditions (Sharkey et al. 2016; IOM 2011). Burn pits, widely used in combat zones before 2009, are open areas for burning solid waste. Burn pit emissions can include harmful particulates and chemicals including dioxins, furans, lead, mercury, volatile organic compounds, and polcyclic aromatic hydrocarbons (Liu et al. 2016).

Despite considerable media coverage and anecdotal reports from concerned service members, epidemiologic evidence of adverse health effects from burn pits exposure has been lacking until recently. For example, as recently as 2014, Abraham et al. noted that “no study has identified an association between burn pit emissions exposure and post deployment chronic lung conditions.” Similarly, the Institute of Medicine concluded in 2011 that there was insufficient evidence to develop firm conclusions about what long-term health effects might be seen in service members exposed to burn pits.

Several factors suggest that this situation is likely to change. First, the number of veterans who have participated in the Airborne Hazards and Open Burn Pit Registry has steadily increased, making it feasible to conduct registry-based epidemiologic studies (Liu et al. 2016). A public law mandated by Congress in in 2013 required the Department of Veterans Affairs to establish a registry for veterans with potential burn pit exposure in Iraq or Afghanistan. Participation in the registry is accomplished by completing its online self-assessment questionnaire, which was designed to obtain a broad picture of the participants’ military exposures and health (Liu et al. 2016).

Secondly, two recent epidemiologic studies have found that exposure to burn pits and deployment to Kabul, Afghanistan is associated with chronic respiratory conditions (Liu et al. 2016; Sharkey et al. 2016). Liu et al. (2016) examined associations between assumed geographic and self-reported burn pit emissions exposure and respiratory and cardiovascular outcomes in participants of the Airborne Hazards and Open Burn Pit Registry. The authors found significant dose-response associations for higher risk of self-reported emphysema, chronic bronchitis, or chronic obstructive pulmonary disease with increased days of deployment within 2 miles of selected burn pits (P-trend = 0.01) and self-reported burn pit smoke exposure (P-trend = 0.0005). Sharkey et al. (2016) conducted a retrospective cohort study to investigate associations between deployment to Kabul, Afghanistan and subsequent respiratory health among U.S. military personnel. The study population consisted of personnel who deployed to Kabul, select Operation Enduring Freedom locations, personnel stationed in the Republic of Korea, and U.S.-stationed personnel. A statistically elevated rate of asthma was observed among personnel deployed to Kabul, relative to U.S.-stationed personnel (IRR 1.61; 95% CI, 1.22–2.12), which is consistent with earlier research findings reported by Szema et al. (2010).

Thirdly, because of the increasing time elapsed since exposure to burn pits during combat, the latency period may be approaching that sufficient to detect associations with emphysema in epidemiologic studies. It may also be feasible to conduct epidemiologic studies to examine associations with malignancies and pulmonary conditions that are infrequent in the general population (for example, glioblastoma of the brain, constrictive bronchiolitis) (King et al. 2011). A proportionate mortality ratio analysis of data from the Burn Pits 360 degrees registry, which was a hypothesis-generating study, found that deaths from malignancies were over-represented compared to deaths from chronic respiratory conditions (unpublished observations, Sunil Halder, 2018).

In addition to additional population-based and registry-based epidemiologic studies of chronic health conditions among veterans exposed to toxic fumes from burn pits, longitudinal studies are needed that include serial measurements of pulmonary function and novel biological markers of exposure to polychlorinated dibeno-p-dioxins/dibenzofurans (Woeller et al. 2016). Individual-level exposure data obtained from microRNAs would reduce misclassification of exposure assessments based upon assumed geographic burn pit emissions exposure and avoid potential recall bias. Other studies are needed that include additional documented burn pit sites integrating particulate matter data, meterological and wind data, and other in-theatre exposures to understand the short- and long-term respiratory health issues associated with open-air burn pit smoke exposure (Smith et al. 2012).
